# Genetic variants within the cancer susceptibility region 8q24 and ovarian cancer risk in Han Chinese women

**DOI:** 10.18632/oncotarget.16861

**Published:** 2017-04-05

**Authors:** Jing Han, Jing Zhou, Hua Yuan, Longbiao Zhu, Hongxia Ma, Dong Hang, Dake Li

**Affiliations:** ^1^ Department of Epidemiology and Biostatistics, School of Public Health, Nanjing Medical University, Nanjing 211166, China; ^2^ Department of Epidemiology, Nanjing Medical University Affiliated Cancer Institute of Jiangsu Province, Nanjing 211166, China; ^3^ State Key Laboratory of Reproductive Medicine, Nanjing Medical University, Nanjing 211166, China; ^4^ Jangsu Key Laboratory of Oral Disease, Nanjing Medical University, Nanjing 210029, China; ^5^ Department of Gynaecology, Jiangsu Provincial Hospital of TCM, Affiliated Hospital of Nanjing University of TCM, Nanjing 210005, China

**Keywords:** ovarian cancer, Han Chinese women, 8q24 region, variant

## Abstract

Accumulating evidence suggests that genetic variants at chromosome 8q24 confer susceptibility to various types of cancer. This case-control study was designed to explore the relationship between genetic variants at 8q24 and ovarian cancer risk in Han Chinese women. Two variants (rs13281615 A > G and rs6983267 T > G) were genotyped in 377 ovarian cancer cases and 1034 cancer-free controls using TaqMan allelic discrimination assay. Logistic regression analysis revealed that the G allele of rs6983267 was significantly associated with increased risk of ovarian cancer (additive model: adjusted OR = 1.21, 95% CI = 1.01–1.43, *P* = 0.048; recessive model: adjusted OR = 1.51, 95% CI = 1.06–2.15, *P* = 0.023). However, no significant association was observed between rs13281615 and ovarian cancer. In stratified analysis, the risk effect of rs6983267 variant remained significant in premenopausal women (additive model: adjusted OR = 1.62, 95% CI = 1.18–2.23, *P* = 0.003). Summarily, this study suggested that 8q24 rs6983267 may contribute to the susceptibility of ovarian cancer in premenopausal Han Chinese women, supporting the pleiotropy of 8q24 in carcinogenesis.

## INTRODUCTION

Ovarian cancer is the leading cause of cancer mortality among global women, with an estimation of 240,000 new cases and 150,000 deaths in 2012 [1]. Up to now, the specific etiology of ovarian cancer remains to be determined. It has been proposed that environmental and behavioral factors may play significant roles in ovarian cancer, such as occupation [2], parity [3], oral contraceptive use [4], and family history of cancer [5]. Furthermore, growing evidence has suggested that genetic variants may also contribute to the risk of ovarian cancer [6].

A segment of 1Mb within chromosome 8q24 has emerged as a susceptibility region for multiple cancers, including prostate, colon, breast, and ovarian cancers, in genome-wide association studies (GWAS) [7–10]. This region contains no known genes but is bounded at its telomeric end by oncogenes *c-MYC* and *PVT1*. Overexpression of *c-MYC* occurs in various types of cancer including ovarian cancer, and reduction of *c-MYC* expression by RNA interference can inhibit tumor growth [11]. An association between *c-MYC* expression and risk allele of rs6983267 at 8q24 was revealed in colorectal cancer. In addition, a noncoding RNA *PVT1* plays an important role in carcinogenesis, and the increase of *PVT1* expression was shown to relate with the GG genotype of rs13281615 at 8q24 [12].

Despite the critical role of 8q24 variants in cancer susceptibility, there has been no study examining the relationship between 8q24 and the risk of ovarian cancer in Chinese women. Therefore, we conducted this case-control study with 377 cases and 1034 healthy controls to explore the association of two potentially functional variants, rs6983267 and rs13281615 at 8q24 with ovarian cancer risk in Han Chinese women.

## RESULTS

Demographic and clinical characteristics of 377 ovarian cancer cases and 1034 cancer-free controls are presented in Table 1. The primary pathological type of cases was epithelial ovarian cancer (86.7%). A total of 103 (27.3%) patients were diagnosed at stage I or II, and 274 (72.7%) were at stage III or IV. The age, occupation, and family history of cancer were comparable between cases and controls (all *P* > 0.05). However, ovarian cancer cases were more likely to have early menarche age, abortion history, and postmenopausal status, but less likely to use oral contraceptives than cancer-free controls (all *P* < 0.05).

**Table 1:** Demographic and clinical characteristics of ovarian cancer cases and controls

Variables	CaseN = 377 (%)	ControlN = 1034 (%)	P^a^
Age, year (mean ± SD)	52.42 ± 12.20	52.75 ± 11.91	0.813
Age at menarche, year (mean ± SD)	14.69 ± 1.50	16.13 ± 1.20	< 0.001
Abortion			< 0.001
Yes	153(40.58)	257(24.85)	
No	204(54.11)	686(66.34)	
Unknown	20(5.31)	91(8.80)	
Menopausal status			< 0.001
Premenopausal	126(33.42)	465(44.97)	
Postmenopausal	224(59.42)	546(52.80)	
Unknown	27(7.16)	23(2.22)	
Occupation			0.433
Farmer	155(41.11)	386(37.33)	
Worker	57(15.12)	167(16.15)	
Other	165(43.77)	481(46.52)	
Oral contraceptive			< 0.001
Yes	213(56.50)	793(76.69)	
No	149(39.52)	217(20.99)	
Unknown	15(3.98)	24(2.32)	
Family history of cancer			0.757
Yes	65(17.24)	190(18.38)	
No	295(78.25)	821(79.40)	
Unknown	17(4.51)	23(2.22)	
Histological type			
Epithelial	327(86.74)		
Other typesb	50(13.26)		
Stage			
I or II	103(27.32)		
III or IV	274(72.68)		

^a^ Student's *t* test and χ^2^ test were used for continuous and categorical variables, respectively

^b^ Other types included germ cell type and sex cord stromal type.

The genotype distributions of rs13281615 and rs6983267 are shown in Table 2. Calling rates of the two variants were above 99% and their genotype frequencies in the control group were consistent with Hardy–Weinberg equilibrium (*P* > 0.05). No obvious linkage disequilibrium between the two variants was detected by LD analysis (D’=0.155, r^2^=0.015). A significant association was observed between rs6983267 and ovarian cancer risk (additive model: adjusted OR = 1.21, 95% CI = 1.01–1.43, *P* = 0.048; recessive model: adjusted OR = 1.51, 95% CI = 1.06–2.15 *P* = 0.023). However, there was no significant association between rs13281615 and ovarian cancer risk.

**Table 2:** Main effects of rs13281615 and rs6983267 on ovarian cancer risk

Genotypes	Case (%)	Control (%)	Crude OR (95% CI)	P	Adjusted OR (95% CI)^a^	P^a^
rs13281615						
AA	86 (23.06)	279 (26.98)	1.00		1.00	
AG	192 (51.47)	503 (48.65)	1.24 (0.92–1.66)	0.153	1.25 (0.92–1.71)	0.159
GG	95 (25.47)	252 (24.37)	1.22 (0.87–1.71)	0.243	1.17 (0.81–1.67)	0.400
Dominant model			1.23 (0.94–1.63)	0.139	1.22 (0.91–1.64)	0.181
Recessive model			1.06 (0.81–1.39)	0.637	1.01 (0.75–1.35)	0.967
Additive model			1.10 (0.94–1.31)	0.242	1.08 (0.90–1.29)	0.397
rs6983267						
TT	128 (33.95)	394 (38.10)	1.00		1.00	
GT	183 (48.54)	510 (49.32)	1.11 (0.85–1.43)	0.456	1.07 (0.81–1.41)	0.631
GG	66 (17.51)	130 (12.57)	1.56 (1.09–2.23)	0.014	1.57 (1.06–2.32)	0.023
Dominant model			1.20 (0.94–1.53)	0.153	1.16 (0.89–1.52)	0.260
Recessive model			1.48 (1.07–2.04)	0.018	1.51 (1.06–2.15)	0.023
Additive model			1.22 (1.03–1.45)	0.026	1.21 (1.01–1.43)	0.048

^a^ Adjusted by age, age at menarche, abortion, menopausal status, and oral contraceptive.

Furthermore, stratified analysis of the associations was performed according to demographic and clinical variables (Table 3 and Supplementary Table 2). The risk effect of rs6983267 on ovarian cancer was shown to be significant in premenopausal women (additive model: adjusted OR = 1.62, 95% CI = 1.18-2.23, *P* = 0.003), with significant heterogeneity in strata of menopausal status (*P* = 0.037).

**Table 3:** Stratified analysis of the association between rs6983267 and ovarian cancer risk

Variables	Case			Control			OR (95% CI) a	Pa	Pb
	TT (%)	GT (%)	GG (%)	TT (%)	GT (%)	GG (%)			
Age, year									
< 52	60 (34.48)	86 (49.43)	28 (16.09)	207 (40.75)	234 (46.06)	67 (13.19)	1.12 (0.85–1.48)	0.414	0.287
≥ 52	68 (33.50)	97 (47.78)	38 (18.72)	187 (35.55)	276 (52.47)	63 (11.98)	1.39 (1.04–1.84)	0.024	
Age at menarche, year									
< 15	56 (32.56)	83 (48.26)	33 (19.19)	82 (36.61)	113 (50.45)	29 (12.95)	1.18 (0.90–1.53)	0.229	0.430
≥ 15	63 (33.69)	93 (49.73)	31 (16.58)	310 (38.41)	396 (49.07)	101 (12.52)	1.40 (1.00–1.94)	0.050	
Abortion									
Yes	52 (33.99)	66 (43.14)	35 (22.88)	78 (30.35)	147 (57.20)	32 (12.45)	1.11 (0.81–1.53)	0.515	0.322
No	68 (33.33)	110 (53.92)	26 (12.75)	281 (40.96)	327 (47.67)	78 (11.37)	1.31 (1.02–1.69)	0.033	
Menopausal status									
Premenopausal	36 (28.57)	65 (51.59)	25 (19.84)	186 (40.00)	221 (47.53)	58 (12.47)	1.62 (1.18–2.23)	0.003	0.037
Postmenopausal	84 (37.50)	105 (46.88)	35 (15.63)	205 (37.55)	270 (49.45)	71 (13.00)	1.05 (0.81–1.35)	0.733	
Occupation									
Farmer	53 (34.19)	77 (49.68)	25 (16.13)	145 (37.56)	200 (51.81)	41 (10.62)	1.23 (0.93–1.63)	0.154	0.121
Worker	14 (24.56)	33 (57.89)	10 (17.54)	76 (45.51)	73 (43.71)	18 (10.78)	1.84 (1.17–2.89)	0.008	
Other	61 (36.97)	73 (44.24)	31 (18.79)	173 (35.97)	237 (49.27)	71 (14.76)	1.07 (0.83–1.37)	0.628	
Oral contraceptive									
Yes	71 (33.33)	104 (48.83)	38 (17.84)	303 (38.21)	392 (49.43)	98 (12.36)	1.23 (0.97–1.56)	0.093	0.970
No	52 (34.90)	70 (46.98)	27 (18.12)	82 (37.96)	108 (50.00)	26 (12.04)	1.24 (0.87–1.75)	0.238	
Family history of cancer									
Yes	21 (32.31)	33 (50.77)	11 (16.92)	64 (33.68)	93 (48.95)	33 (17.37)	1.09 (0.61–1.93)	0.774	0.593
No	103 (34.92)	140 (47.46)	52 (17.63)	322 (39.22)	405 (49.33)	94 (11.45)	1.29 (1.03–1.61)	0.027	

^a^ Adjusted for age, age at menarche, abortion, menopausal status, and oral contraceptive where appropriate in additive models.

^b^ *P* for heterogeneity test.

## DISCUSSION

In the current study, we investigated the relationship between two variants (rs6983267 and rs13281615) at 8q24 identified by the GWAS of multiple cancers and ovarian cancer risk. We revealed that the G allele of rs6983267 was significantly associated with increased risk of ovarian cancer in premenopausal Han Chinese women.

Chromosomal 8q24 has been considered the most important susceptibility region for various types of malignancy, including prostate [7, 13], colorectal [14–16], and breast cancer [9, 17]. However, the mechanism by which 8q24 variants affect cancer susceptibility is not fully understood. None of identified variants reside within known genes, of which there are few across 8q24. Functional evidence has indicated that this risk region may act as a regulatory hub by physical interactions with several neighboring genes important for carcinogenesis such as *c-Myc* and *PVT1* [18]. Oncogene *c-Myc* encodes phosphoproteins that participate in the regulation of cell proliferation, apoptosis, and differentiation [19]. Previous studies demonstrated that gene amplification and increased expression of *c-Myc* could promote the development of ovarian cancer [20, 21]. PVT1 is located adjacent to *c-MYC* and functions as a noncoding RNA with many alternatively spliced isoforms. Both *PVT1* copy number gains and overexpression have been implicated in the development of many tumors, including ovarian cancer [22]. Small interfering RNA–mediated reduction in either *PVT1* or *MYC* expression can inhibit cellular proliferation of ovarian cancer [22].

The variant rs6983267 has been associated with both prostate and colorectal cancers in different ethnic populations [16, 23–25]. It is located far away from any coding sequences, and the nearest gene is *c-MYC*. Functional studies suggest that rs6983267 is located in a transcriptional enhancer and exhibits long-range physical interaction with *c-Myc* [26]. The variant has enhancer-related histone marks and can form a 335-kb chromatin loop to interact with the c-MYC promoter, resulting in elevated *c-Myc* expression [27]. In addition, transpiration factors such as *TCF4* may bind preferentially to the risk allele G of rs6983267, enhancing the responsiveness to Wnt signaling [28]. These findings provide a potential mechanism for the source of association between rs6983267 and ovarian cancer. However, additional studies are required to prove the interaction of rs6983267 with *c-MYC* and other transcription factors in the pathogenesis of ovarian cancer.

Previous studies identified rs13281615 at 8q24 as an independent susceptibility locus for breast cancer [17, 29]. The GG genotype of rs13281615 was significantly associated with estrogen receptor positivity, higher tumor grade and higher proliferation index in breast cancer. In addition, *PVT1* expression was elevated in breast cancer tissues and the increase was related with the GG genotype of rs13281615 [12]. Overexpression of *PVT1* has been demonstrated in in a variety of cancer types including ovarian cancer [30]. Increased expression of *PVT1* in ovarian cancer cells may promote cisplatin resistance by regulating apoptotic pathways [31]. In the current study, we did not find significant association between rs13281615 and ovarian cancer risk in Han Chinese women. However, insufficient statistical power due to relatively small sample size in our study may also account for the lack of association. Independent studies with larger sample size are needed to validate our results.

Several limitations need to be mentioned. First, for lacking of samples, we conducted only one stage case-control study. Second, the biological function of rs6983267 in ovarian cancer was not further investigated in this study. Despite these limitations, this study was the first to provide evidence that rs6983267 at 8q24 may contribute to the risk of ovarian cancer in Han Chinese women. Replication studies in diverse populations and functional analyses are warranted to confirm the role of 8q24 variants in ovarian cancer.

## MATERIALS AND METHODS

### Study population

This study was approved by the institutional review board of Nanjing Medical University, and each participant signed an informed consent before the enrollment. Briefly, a total of 377 patients with ovarian cancer were recruited from the areas of relatively high incidence, including cities of Nantong, Wuxi and Nanjing in east China's Jiangsu province, as previously described [32]. All ovarian cancer cases had definite histopathological diagnosis and met the following inclusion criteria: (1) Han Chinese; (2) local residences (at least 5 years); (3) without tumor history in any other organs.

Cancer-free controls were randomly selected from a community-based cohort of over 30,000 participants for non-infectious disease screening program in Jiangsu Province, during the same period as the cases were recruited [33]. The controls were genetically unrelated Han Chinese women, having a local residence (at least 5 years) and no history of cancer. A one-on-one interview was carried out by a trained interviewer to collect demographic information, age at menarche, menstrual and reproduction history, and environmental exposures. Approximately 5 ml of venous blood was obtained from each participant. Eventually, 377 ovarian cancer cases and 1034 cancer-free controls frequency-matched by age (5-year interval) were included in this study.

### Genotyping

Genomic DNA was extracted from leukocyte pellets by traditional proteinase K digestion, followed by phenol-chloroform extraction and ethanol precipitation. Variants were genotyped using the TaqMan allelic discrimination assay on ABI PRISM 7900 HT platform (Life Technologies, CarIsbad, USA). Information of the primers and probes was shown in Supplementary Table 1. The genotyping was performed without knowing the subjects’ case or control status. In each 384-well reaction plate, two negative controls were added for quality control. To make sure the reproducibility of genotyping, 50 cases and 50 controls were randomly selected to be retested, yielding a 100% concordance.

### Statistical analysis

Differences in demographic characteristics, exposure variables, and genotypes frequencies between the cases and controls were evaluated by the χ^2^ test for categorical variables and Student's t test for continuous variables. Hardy-Weinberg equilibrium (HWE) was tested using goodness-of-fit χ^2^ test. Haploview was employed to analyze linkage disequilibrium (LD) parameters (i.e., D′ and r^2^). The association between variants and ovarian cancer risk was estimated by computing odds ratios (ORs) and their 95% confidence intervals (CIs) from logistic regression analysis in three genetic models (additive, dominant, and recessive). Each model makes different assumptions about the genetic effect, as previously described [34]. Age and statistically significant variables in univariate analysis were included in multivariate analysis. In stratified analysis, the χ^2^-based *Q* test was used to assess the heterogeneity of associations between subgroups. All statistical analyses were performed with Statistical Analysis System software (v9.1.3, SAS Institute, Cary, NC). A two-sided *P* value of less than 0.05 was used as the criterion for statistical significance.

FSFA

## SUPPLEMENTARY MATERIALS TABLES


