# Supplementary material for: Genetic variants within the cancer susceptibility region 8q24 and ovarian cancer risk in Han Chinese women
Source: Oncotarget. 2017 Apr 5;8(22):36462–8. doi: 10.18632/oncotarget.16861 (PMC5482668; doi:10.18632/oncotarget.16861)
Supplement: Supplementary file 1 [file oncotarget-08-36462-s001.pdf]

Genetic variants within the cancer susceptibility region 8q24 and ovarian cancer risk in Han Chinese women

Supplementary Materials

Supplementary Table 1: Information of primers and probes for TaqMan allelic discrimination assay

Variants		Sequence (5′-3′)
rs13281615	Primer	F:TAATGGAGAAGCACTATCCTGG
		R:GCAGCAGATAGGAGGAATCAC
	Probe	A: CATCAAAAGAAAGCAGAACGCAGA
		G: CATCAAAAGAAGGCAGAACGCAG
rs6983267	Primer	F:CGAATAAACTCTCCTCCTACCAC
		R:GGTTCCTGCCCTTTGATTC
	Probe	C: CTCAGTGCCTTTCA
		A: CTCAGTGACTTTCAT

Supplementary Table 2: Stratified analysis of the association between rs13281615 and ovarian cancer risk

Variables	Case			Control			OR (95% CI)[a]	*P* [a]	*P* [b]
	AA (%)	AG (%)	GG (%)	AA (%)	AG (%)	GG (%)			
Age, year									
< 52	39 (22.41)	89 (51.15)	46 (26.44)	135 (26.57)	249 (49.02)	124 (24.41)	0.94 (0.72–1.22)	0.638	0.496
≥ 52	47 (23.62)	103 (51.76)	49 (24.62)	144 (27.38)	254 (48.29)	128 (24.33)	1.07 (0.82–1.39)	0.642	
Age at menarche, year									
< 15	42 (24.85)	85 (50.30)	42 (24.85)	52 (23.21)	118 (52.68)	54 (24.11)	1.12 (0.88–1.43)	0.362	0.162
≥ 15	40 (21.86)	94 (51.37)	49 (26.78)	225 (27.88)	384 (47.58)	198 (24.54)	0.84 (0.61–1.16)	0.293	
Abortion									
Yes	37 (24.34)	82 (53.95)	33 (21.71)	66 (25.68)	120 (46.69)	71 (27.63)	0.93 (0.69–1.26)	0.637	0.469
No	46 (22.89)	99 (49.25)	56 (27.86)	194 (28.28)	332 (48.40)	160 (23.32)	1.07 (0.85–1.35)	0.578	
Menopausal status									
Premenopausal	28 (22.40)	69 (55.20)	28 (22.40)	129 (27.74)	237 (50.97)	99 (21.29)	1.02 (0.75–1.38)	0.904	0.960
Postmenopausal	53 (23.98)	108 (48.87)	60 (27.15)	140 (25.64)	258 (47.25)	148 (27.11)	1.01 (0.80–1.27)	0.962	
Occupation									
Farmer	33 (21.43)	82 (53.25)	39 (25.32)	103 (26.68)	193 (50.00)	90 (23.32)	1.17 (0.87–1.57)	0.303	0.373
Worker	11 (19.64)	28 (50.00)	17 (30.36)	52 (31.14)	79 (47.31)	36 (21.56)	1.02 (0.59–1.78)	0.941	
Other	42 (25.77)	82 (50.31)	39 (23.93)	24 (25.78)	231 (48.02)	126 (26.20)	0.87 (0.65–1.16)	0.345	
Oral contraceptive									
Yes	43 (20.48)	110 (52.38)	57 (27.14)	223 (28.12)	384 (48.42)	186 (23.46)	1.14 (0.91–1.43)	0.257	0.103
No	41 (27.70)	73 (49.32)	34 (22.97)	50 (23.15)	104 (48.15)	62 (28.70)	0.82 (0.59–1.13)	0.226	
Family history of cancer									
Yes	13 (20.31)	37 (57.81)	14 (21.88)	45 (23.68)	98 (51.58)	47 (24.74)	0.86 (0.47–1.56)	0.620	0.481
No	69 (23.63)	143 (48.97)	80 (27.40)	230 (28.01)	392 (47.75)	199 (24.24)	1.08 (0.88–1.32)	0.481	

[a]Adjusted for age, age at menarche, abortion, menopausal status, and oral contraceptive where appropriate in additive models.
[b]*P* for heterogeneity test.
